# Inorganic and *Erythroxylum coca* Leaf Extract-Mediated Synthesis of Gold Nanoparticles: A Comparative Study of Size, Surface Chemistry, and Colloidal Stability

**DOI:** 10.3390/nano16060341

**Published:** 2026-03-10

**Authors:** Juan A. Ramos-Guivar, Henry Daniel Lizana-Segama, Mercedes del Pilar Marcos-Carrillo, Noemi-Raquel Checca-Huaman

**Affiliations:** 1 Grupo de Investigación de Nanotecnología Aplicada para Biorremediación Ambiental, Energía, Biomedicina y Agricultura (NANOTECH), Facultad de Ciencias Físicas, Universidad Nacional Mayor de San Marcos, Av. Venezuela Cdra 34 S/N, Ciudad Universitaria, Lima 15081, Peru; 2 Centro Brasileiro de Pesquisas Físicas (CBPF), R. Xavier Sigaud, 150, Urca, Rio de Janeiro 22290-180, Brazil

**Keywords:** gold nanoparticles, biosynthesis, plant extract, colloidal stability, surface chemistry, *Erythroxylum coca*

## Abstract

Gold nanoparticles (AuNPs) were synthesized via two complementary routes, an inorganic surfactant-mediated method and a plant-extract-assisted biosynthesis, to elucidate how synthesis pathways influence nanoparticle physicochemical properties. In the inorganic route, hexadecyltrimethylammonium bromide (CTAB)-stabilized AuNPs were prepared using CTAB dissolution temperatures of 70–90 °C. UV–Vis spectroscopy showed localized surface plasmon resonance (LSPR) bands at 554–556 nm, while dynamic light scattering (DLS) indicated a decrease in hydrodynamic diameter from 110 to 97 nm with increasing dissolution temperature. Zeta potentials above +40 mV indicated strong electrostatic stabilization, and transmission electron microscopy (TEM) revealed ultrasmall Au cores with a narrow size distribution (2.4–3.0 nm) and a face-centered cubic crystal structure. In the biosynthetic route, AuNPs were obtained using aqueous *Erythroxylum coca* leaf extracts (1–4% *w*/*v*). The extracts exhibited a concentration-dependent red shift (~380 to ~420 nm), and biosynthesized AuNPs displayed LSPR bands in the 550–580 nm range. DLS yielded hydrodynamic diameters of 270–390 nm, with pronounced aggregation (3341 nm) at the lowest extract concentration. Under optimized conditions (HC5, n = 5), reproducible plasmonic and colloidal properties were obtained (maximum absorbance, localized surface plasmon resonance wavelength (λ_max_) = 569.6 ± 1.7 nm; hydrodynamic diameter (*D*_H_) = 237.6 ± 24.3 nm; absolute zeta potential (|ζ|)= 32.2 ± 2.6 mV). TEM analysis indicated predominantly quasi-spherical particles with a broader, log-normal size distribution, consistent with extract-mediated growth under heterogeneous organic capping environments.

## 1. Introduction

AuNPs exhibit size-, morphology-, and surface-dependent optical, electronic, and catalytic properties, primarily governed LSPR phenomena. The position, intensity, and bandwidth of the LSPR band are highly sensitive to nanoparticle size distribution, crystallinity, interparticle spacing, and surface chemistry. In addition, electrostatic stabilization and long-term colloidal robustness are critically influenced by surface charge density and interfacial molecular organization. Consequently, precise control over synthetic parameters, such as reducing environment, temperature, surfactant assembly state, and reactant concentration, is essential to tailor AuNP physicochemical behavior for applications in sensing, catalysis, biomedicine, and environmental remediation [1,2,3].

Conventional chemical reduction methods have been widely employed to achieve reproducible control over AuNP nucleation and growth. These approaches typically rely on strong reducing agents and surfactants that act as stabilizers and shape-directing agents, enabling modulation of kinetic and thermodynamic growth pathways [4,5]. Among these systems, CTAB has been extensively used as a cationic surfactant due to its ability to form organized micellar and bilayer assemblies at nanoparticle interfaces [3,6,7,8,9]. The interfacial assembly state of CTAB plays a decisive role in governing reduction kinetics, electrostatic stabilization, anisotropic growth pathways, and hydrodynamic behavior. Variations in CTAB concentration, dissolution temperature, and micellar organization have been reported to significantly influence nanoparticle morphology and colloidal stability [7,8,9]. However, residual CTAB adsorption may affect long-term dispersion behavior and biological interactions, as CTAB has been shown to exhibit measurable cytotoxic effects and persistent surface contamination in mammalian systems [10]. These limitations have stimulated growing interest in alternative synthetic strategies that reduce reliance on synthetic surfactants while maintaining control over nanoparticle physicochemical properties.

In recent years, plant-extract-mediated biosynthesis has emerged as a sustainable alternative to conventional surfactant-based methods. Plant-derived metabolites—including polyphenols, flavonoids, alkaloids, and organic acids—can simultaneously act as reducing and stabilizing agents, enabling nanoparticle formation under mild conditions without synthetic surfactants [11,12,13,14]. Unlike CTAB-mediated systems, biosynthetic environments introduce chemically heterogeneous organic coronas that may alter reduction kinetics, surface charge distribution, nucleation regimes, and anisotropic growth behavior. The multiplicity of functional groups present in plant extracts enables synergistic reduction pathways and dynamic surface passivation processes, which may generate broader size distributions and distinct optical signatures compared to inorganic systems.

Despite numerous reports on green synthesis of AuNPs, systematic studies that quantitatively correlate extract concentration with key physicochemical parameters, such as hydrodynamic size distribution, zeta potential, LSPR position, reduction kinetics, and long-term colloidal stability, remain limited. Furthermore, direct comparisons between surfactant-controlled inorganic systems and alkaloid-rich plant-mediated biosynthetic routes within a unified experimental framework are scarce. In particular, the influence of extract concentration on anisotropic growth behavior and plasmonic response has not been thoroughly evaluated in parallel with surfactant dissolution conditions.

*Erythroxylum coca* leaves present a chemically diverse phytochemical profile containing tropane alkaloids, phenolic compounds, flavonoids, and oxygen-containing functional groups capable of coordinating metal ions and facilitating electron transfer processes. The coexistence of amine, hydroxyl, carbonyl, and aromatic moieties provides multiple potential pathways for Au^3+^ reduction and surface stabilization. This chemically rich composition makes *E. coca* extract a particularly suitable model system for investigating synergistic reduction mechanisms, heterogeneous surface passivation, and potential anisotropic nanoparticle growth under biologically mediated conditions.

Against this background, the present study establishes a systematic comparative framework between CTAB-mediated inorganic synthesis and *Erythroxylum coca* leaf extract-mediated biosynthesis of AuNPs. The primary objectives are (i) to quantify how CTAB dissolution temperature and plant-extract concentration modulate AuNPs optical response (LSPR position and band shape), hydrodynamic diameter, zeta potential, and core-size distributions, and (ii) to determine the conditions that maximize colloidal reproducibility and long-term stability in each route. By systematically varying these parameters under controlled conditions, a direct structure–property comparison is enabled between inorganic surfactant-controlled systems and organic extract-mediated systems, providing mechanistic insight into how reaction environment and interfacial stabilization govern nanoparticle formation and stability, with implications for application-oriented and sustainability-driven synthesis design.

## 2. Materials and Methods

To conduct synthesis experiments, tetrachloroauric acid, H[AuCl_4_]·3H_2_O (with 99.9% purity), sodium borohydride, NaBH_4_ (with 99% purity), and CTAB (with 98% purity), were purchased from Sigma Aldrich (St. Louis, MO, USA) for all the reagents.

## 2.1. Solutions Preparation

They were prepared as a precursor solution of chloroauric acid (HAuCl_4_) at a concentration of 0.250 mM in ultrapure water. As a reducing agent, sodium borohydride, NaBH_4_, was used at a concentration of 17 mM in ultrapure water at a temperature of 1 °C. The surfactant CTAB was dissolved in ultrapure water in a 10 mL flask with a cap at a concentration of 17 mM. In the dissolution of CTAB, a magnetic stirrer (450 RPM) was used at temperatures of 70 °C, 80 °C, and 90 °C, varying the time between 20 min and 40 min. Ultrapure water was obtained from the PURELAB Quest ELGA (High Wycombe, United Kingdom) purification system with a resistivity of 18.2 MΩ·cm.

It is important to emphasize that the temperatures of 70–90 °C refer exclusively to the pre-dissolution step of CTAB prior to nanoparticle synthesis. These temperatures were used to control the surfactant assembly state before its introduction into the reaction medium. The subsequent gold reduction reaction itself was conducted separately at a fixed temperature of 100 °C, as described in Section 2.2.

### 2.2. Inorganic Synthesis of AuNPs

20 mL of the precursor solution was placed in a 50 mL Erlenmeyer flask and heated under magnetic stirring (450 RPM) until reaching the boiling point (100 °C). This temperature corresponds to the reduction stage and is independent of the CTAB pre-dissolution temperature described previously. Once the indicated temperature was reached, 1 mL of CTAB was added, followed by 1 mL of NaBH_4_. During the 20 min of synthesis, the initial volume was controlled by adding ultrapure water, then it was cooled in a water bath in a 250 mL beaker until reaching room temperature (27 °C). A total of six samples were synthesized, all with the same amount and concentration of reagents ([HAuCl_4_] = 0.250 mM, [NaBH_4_] = 17 mM and [CTAB] = 17 mM). The difference between them lies in the CTAB dissolution time, which was 20 min for samples H13, H14, and H15, and 40 min for samples H13b, H14b, and H15b, as well as being carried out at different temperatures (Table 1).

The samples were stored in sealed glass vials at room temperature (25 ± 2 °C), protected from direct light exposure and without agitation during the 30-day stability evaluation.

### 2.3. Preparation of the Extract

Coca leaves (*Erythroxylum coca*) were carefully selected to ensure that they were intact and free from stains, rot, or any signs of deterioration. The selected leaves were washed with ultrapure water to remove surface impurities and subsequently dried in an oven at 70 °C for 24 h. After drying, the leaves were crushed or cut into small pieces to facilitate weighing and handling. Dried *Erythroxylum coca* leaves were purchased from a local vendor at Bolívar Market (Pueblo Libre, Lima, Peru) in September 2025 and stored at 25 °C until extract preparation.

Using the processed plant material, extracts with different concentrations were prepared according to the weight/volume percentage (% *w*/*v*). For this purpose, 1 g (1% *w*/*v*), 1.5 g (1.5% *w*/*v*), 2 g (2% *w*/*v*), 2.5 g (2.5% *w*/*v*), 3 g (3% *w*/*v*), 3.5 g (3.5% *w*/*v*), and 4 g (4% *w*/*v*) of chopped coca leaves were weighed, each intended to obtain an extract at a distinct concentration.

Each weighed mass was placed into a flat-bottom glass flask containing 100 mL of ultrapure water, and the extraction was carried out by decoction through boiling for 5 min. After completion of the decoction process, the mixture was gradually cooled in a water bath until reaching room temperature (300 K). Once cooled, the extract was subjected to vacuum filtration using Whatman filter paper with a pore size of 11 µm to remove suspended solids.

**Scheme 1:**
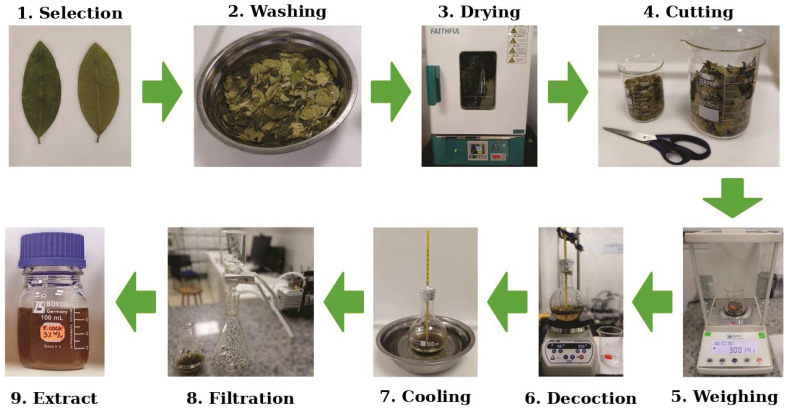
Step-by-step preparation of *Erythroxylum coca* leaf aqueous extract employed for the biosynthesis of AuNPs. The process includes (1) selection of intact leaves, (2) washing with ultrapure water, (3) oven drying at 70 °C, (4) cutting into small pieces, (5) weighing of the plant material, (6) decoction by boiling in ultrapure water, (7) cooling to room temperature, (8) vacuum filtration to remove solid residues, and (9) collection of the final aqueous extract for subsequent use.

The resulting filtrate (100 mL) was collected and stored in a graduated glass bottle with a screw cap, previously washed and sterilized. Finally, the seven flasks were properly labeled and stored at 5 °C until further use (Scheme 1).

### 2.4. Biosynthesis of AuNPs

The biosynthesis of AuNPs was carried out using coca leaf extract as both a reducing and stabilizing agent. The extract was previously prepared at a specific concentration expressed as % *w*/*v* (weight/volume), which was used as the added volume during the biosynthesis process.

A 1 mM solution of chloroauric acid (HAuCl_4_) was employed as the metallic precursor. The synthesis was conducted in a borosilicate glass flask, selected due to its thermal and chemical resistance, making it suitable for chemical reactions. Initially, 20 mL of the gold precursor solution were placed in the flask, followed by the addition of 4 mL of coca leaf extract at the predetermined % *w*/*v* concentration, resulting in an initial total volume of 24 mL.

The reaction mixture was maintained under magnetic stirring at 550 rpm at room temperature (≈300 K) for 24 h, with continuous exposure to laboratory light, a condition required to promote the reduction of the gold precursor (Table 2). After completion of the 24 h reaction period, the suspension was subjected to centrifugation at 4000 rpm for 15 min. This washing procedure was repeated twice to purify the sample. During this process, the initial pH of the reaction mixture was approximately 5, increasing to 6.5 after the second washing step.

**Scheme 2:**
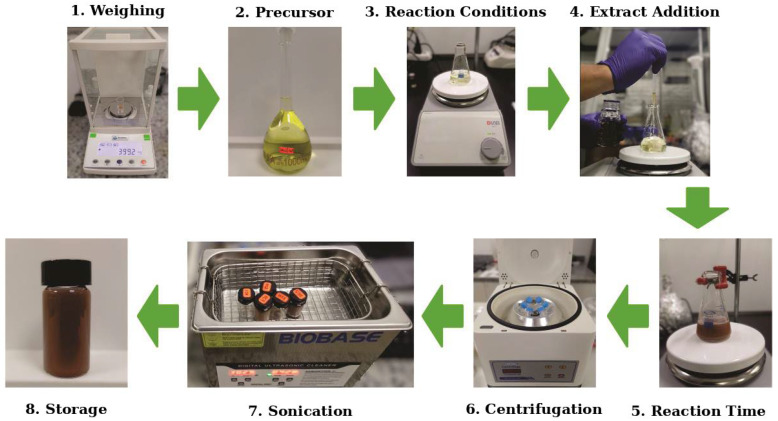
Schematic representation of the biosynthesis process of AuNPs using *Erythroxylum coca* leaf extract. The procedure involves (1) weighing of the reagents, (2) preparation of the gold precursor solution, (3) establishment of reaction conditions under magnetic stirring, (4) addition of the coca leaf extract, (5) reaction time under controlled conditions, (6) centrifugation for nanoparticle recovery and washing, (7) sonication to redisperse the AuNPs, and (8) storage of the final AuNP suspension for subsequent characterization.

The centrifuged material (solid fraction containing the AuNPs) was carefully redispersed in ultrapure water and sonicated for 20 min to obtain a homogeneous, aggregate-free suspension. Finally, the resulting volume (24 mL) was stored in labeled, screw-cap glass vials (seven vials) at room temperature. All vials were previously sterilized and properly labeled for subsequent use (Scheme 2).

Finally, five independent biosynthesis experiments were performed under identical conditions to evaluate reproducibility.

### 2.5. Characterization

The optical properties of the synthesized AuNPs were analyzed using UV–visible spectroscopy. Absorbance spectra were recorded over a wavelength range of 300–1000 nm using an Avantes spectrometer (Avantes B.V., Apeldoorn, The Netherlands). The measurements were performed at room temperature using plastic cuvettes, and the LSPR band was used to monitor nanoparticle formation and assess changes in particle size distribution and aggregation behavior. The LSPR peak parameters were extracted from the Gaussian fits using the OriginPro 9.0 software.

The hydrodynamic diameter and surface charge of the colloidal AuNPs were determined by DLS and electrophoretic mobility measurements, respectively. These analyses were carried out using a Brookhaven NanoBrook 90 Plus PALS instrument (Brookhaven Instruments Corporation, Nashua, NH, USA). The Particle Solutions software (BIC v.3.6.0.7122) was employed for data acquisition and analysis. Hydrodynamic size distributions were obtained from DLS measurements, while the zeta potential values were calculated from electrophoretic mobility using the Smoluchowski approximation. The reported zeta potential values are the mean of ten independent measurements. These measurements provided information on colloidal stability and surface charge characteristics of the AuNP suspensions.

TEM imaging was used to evaluate nanoparticle morphology and to extract metallic core size distributions. Samples were prepared by depositing a small aliquot (e.g., 5–10 µL) of a diluted AuNP suspension onto carbon-coated copper grids (e.g., lacey carbon film, 300 mesh), followed by drying under ambient conditions prior to imaging. Micrographs were acquired at multiple magnifications, using scale bars ranging from 50 to 200 nm, ensuring clear identification of individual AuNPs for measurement. Particle size distribution (PSD) histograms were determined by analyzing 20–25 micrographs per sample and counting ~800–1200 individual nanoparticles (depending on sample) using ImageJ (v1.54g). The resulting PSDs were modeled using a log-normal distribution, from which the characteristic diameter and dispersion parameters were obtained. TEM-based polydispersity was estimated from the fitted distribution (e.g., via the normalized width/standard deviation of the log-normal fit), and represents the dispersion of metallic core sizes in the dried state, distinct from the DLS-derived hydrodynamic size dispersion in suspension.

## 3. Results and Discussion

### 3.1. Synthesis of AuNPs at Different CTAB Dissolution Temperatures in a Time of 20 min

**Figure 1:**
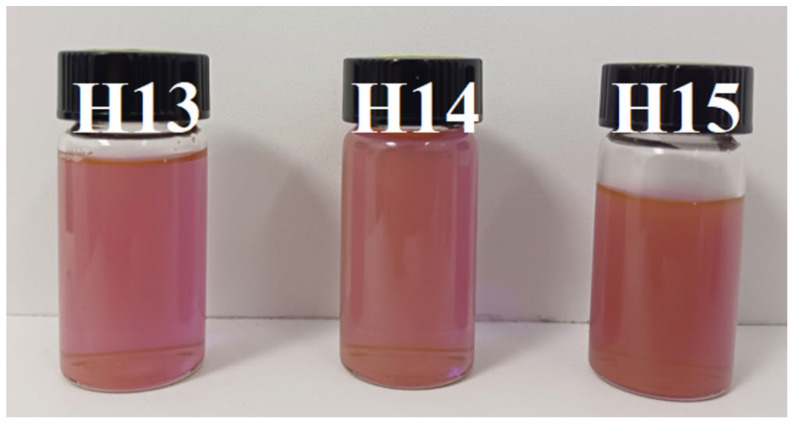
Samples at 20 min of CTAB dissolution: (H13) 70 °C, (H14) 80 °C, and (H15) 90 °C.

In Figure 1, the samples H13, H14, and H15 can be observed; at first glance, a color between red and yellow for all the samples. This solution darkens as the temperature of the solution increases. A month after the synthesis, there was no sedimentation of the samples, indicating the colloidal stability of the AuNPs.

**Figure 2:**
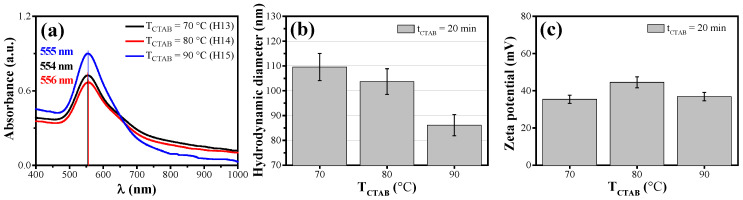
Samples H13 (70 °C), H14 (80 °C) and H15 (90 °C) at 20 min of CTAB dissolution. At different temperatures of CTAB: (**a**) UV-Vis absorbance spectra, (**b**) hydrodynamic diameters, and (**c**) zeta potential values. In panel (**a**), the colored vertical lines mark the wavelength of the absorbance maximum for each sample, evidencing the slight shift of the LSPR band among H13, H14, and H15.

UV–Vis spectra confirmed AuNP formation, and the LSPR bands of H13–H15 showed no clear differences, consistent with predominantly spherical AuNPs. A maximum wavelength of 554, 556, and 555 nm was observed, respectively, indicating that there was no shift in the position of the absorbance band (nm) as the temperature of the CTAB solution increased (Figure 2). The opposite occurs in other studies; the shift in the maximum absorbance wavelength increases with higher synthesis temperatures [15,16].

An inverse trend was observed between CTAB dissolution temperature and hydrodynamic diameter for the 20 min dissolution series: H13, H14, and H15 exhibited hydrodynamic diameters of 110, 104, and 97 nm, respectively (Figure 2). This suggests that the CTAB dissolution temperature modulates the surfactant assembly state and, consequently, the effective hydrodynamic size of the CTAB-stabilized AuNP dispersions, with H15 showing the smallest value. The observed decrease in hydrodynamic diameter with increasing CTAB dissolution temperature is consistent with the temperature-dependent self-assembly of CTAB and its interfacial adsorption dynamics. At higher dissolution temperatures, CTAB aggregates can reorganize and dissolve more uniformly, increasing the availability of surfactant monomers/micelles that rapidly adsorb onto newly formed Au nuclei. This modifies the extent and homogeneity of surface coverage during nucleation and growth, which directly impacts the effective colloidal entity measured by DLS (Au core + CTAB corona + associated micellar structures). Moreover, NaBH_4_ reduction of Au^3+^ is highly kinetic and occurs within a microenvironment shaped by CTAB assemblies; changes in micellar structuring and adsorption dynamics can alter local transport, nucleation density, and growth pathways. Therefore, the hydrodynamic size trends should be interpreted as arising from the coupled effects of temperature-dependent CTAB micellar structuring, surfactant adsorption at the nanoparticle interface, and NaBH_4_-driven reduction kinetics, rather than solely from a generic “stabilizing role” of CTAB [3,7]. To produce uniform and large core–shell AuNPs, meticulous temperature control is required during the formation not only before the synthesis but also during the synthesis as in gold seeds [17].

Figure 2 shows consistently high positive zeta potentials for H13–H15 (≥+40 mV), consistent with strong electrostatic stabilization at all CTAB dissolution temperatures [18]. Among them, H14 presents the highest zeta potential, indicating comparatively stronger colloidal stability.

Among the 20 min dissolution conditions, H15 combines the smallest hydrodynamic diameter with a high positive zeta potential, suggesting the most stable dispersion within this set.

### 3.2. Monitoring over Time of the Absorbance Band, Hydrodynamic Diameter, Zeta Potential, and for Samples with a Dissolution Time of 20 min

**Figure 3:**
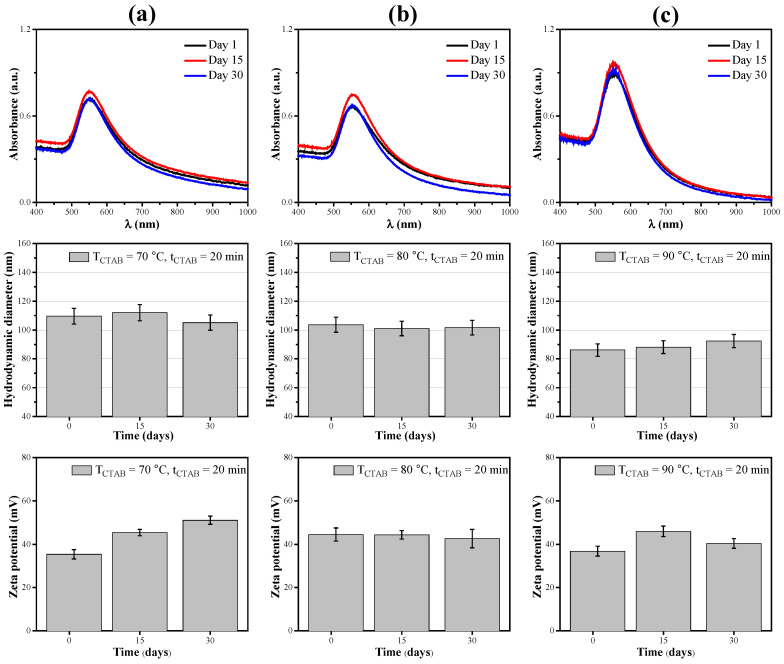
Temporal monitoring of UV-Vis absorbance spectra, hydrodynamic diameters, and zeta potential values for samples (**a**) H13, (**b**) H14 and (**c**) H15.

For H13, H14, and H15, the LSPR band remains essentially unchanged over the 30-day monitoring period, suggesting preservation of the optical signature and no major changes in the dominant nanoparticle population. Consistently, the hydrodynamic diameter stays within ~104–112 nm, and the zeta potential remains strongly positive (+35 to +50 mV), indicating sustained electrostatic stabilization. A slight shift in the measured zeta potential toward more positive values was observed over time (Figure 3). This trend should not be interpreted as the AuNPs “carrying more charge,” but rather as a change in the interfacial electrokinetic potential associated with the CTAB-coated interface and the surrounding electrical double layer. Such variations may arise from subtle changes in ionic strength (double-layer compression/relaxation), counterion association, or CTAB adsorption–desorption/rearrangement at the nanoparticle surface, while the colloid remains electrostatically stabilized. This is corroborated by other studies which determined that the main growth of the particles occurs through the Ostwald ripening mechanism, although the presence of particle fusion was also identified. On the other hand, while the initial particles exhibit a spherical shape, the larger particles develop a variety of morphologies, including spheres, bipyramids, decahedra, deca-tetrahedra, triangular plates, and rod-like structures [19].

### 3.3. Synthesis of AuNPs at Different CTAB Dissolution Temperatures in a Time of 40 min

**Figure 4:**
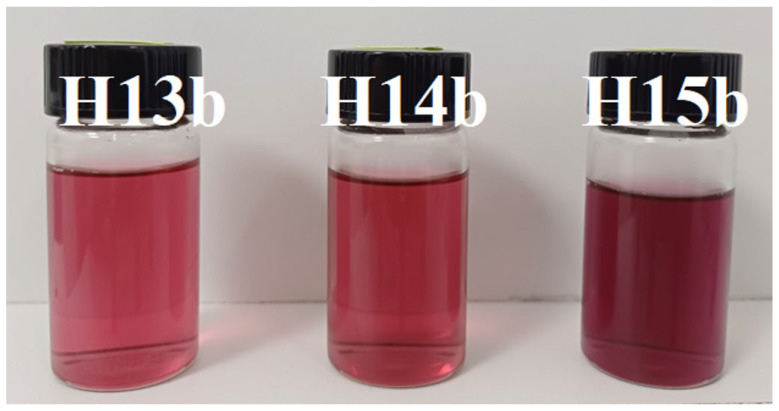
Samples at 40 min of CTAB dissolution: (H13b) 70 °C, (H14b) 80 °C, and (H15b) 90 °C.

For samples H13b, H14b, and H15b, a color ranging from violet to red for all the samples was observed. The suspensions darkened as the dissolution temperature increased. One month after synthesis, there was no sedimentation of the samples, indicating the stability of the AuNPs (Figure 4).

**Figure 5:**
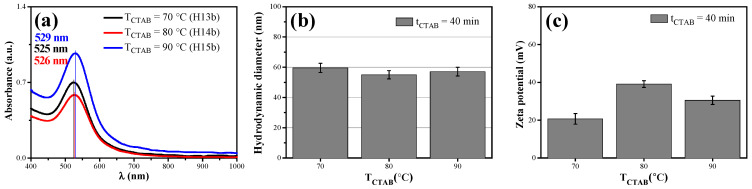
Samples H13b (70 °C), H14b (80 °C), and H15b (90 °C) at 40 min of CTAB dissolution. At different temperatures of CTAB: (**a**) UV-Vis absorbance spectra, (**b**) hydrodynamic diameters, and (**c**) zeta potential values. In panel (**a**), the colored vertical lines mark the wavelength of the absorbance maximum for each sample, evidencing the slight shift of the LSPR band among H13b, H14b, and H15b.

In Figure 5, the UV-Vis absorbance bands helped us identify the AuNPs in the solution immediately. There is no relationship between the absorbance bands and the CTAB dissolution temperature for samples H13b, H14b, and H15b, characteristic of spherical AuNPs. Their maximum wavelength was found at 525 nm, 526 nm, and 529 nm, respectively, indicating a slight shift to the right as the dissolution temperature increases. This indicates that CTAB played a fundamental role in the growth of AuNPs [20].

The DLS measurements show no clear dependence of the hydrodynamic diameter on the CTAB dissolution temperature. The hydrodynamic diameter for samples H13b, H14b, and H15b was 60 nm, 55 nm, and 57 nm, respectively (Figure 5). Indicating that there is a rapid and controlled synthesis due to the stabilizing property of CTAB with a dissolution time of 40 min, where the sample H14b has the smallest hydrodynamic diameter [16]. The weak dependence of hydrodynamic diameter on dissolution temperature at 40 min suggests that prolonged dissolution may produce a more equilibrated CTAB self-assembly state prior to reaction, reducing variability in micellar structuring and promoting more consistent adsorption dynamics during NaBH_4_ reduction. Under these conditions, the CTAB-controlled interfacial environment likely becomes more uniform, diminishing temperature-driven differences in the effective hydrodynamic size measured by DLS [3,7].

In Figure 5, samples H13b, H14b, and H15b exhibit positive zeta potentials, reaching values of approximately +40 mV at 80 °C. Across the tested dissolution temperatures, the zeta potential remains within a range consistent with electrostatically stable dispersions [19]. Among these conditions, H14b shows the highest (or near-highest) zeta potential, suggesting comparatively stronger colloidal stabilization under that condition.

Among the CTAB samples prepared with 40 min dissolution, H14b exhibits the smallest hydrodynamic diameter together with a narrow DLS size distribution and a strongly positive zeta potential, suggesting the most stable colloidal behavior within this series compared with H13b and H15b.

### 3.4. Monitoring over Time of the Absorbance Band, Hydrodynamic Diameter, Zeta Potential, and for Samples with a Dissolution Time of 40 min

**Figure 6:**
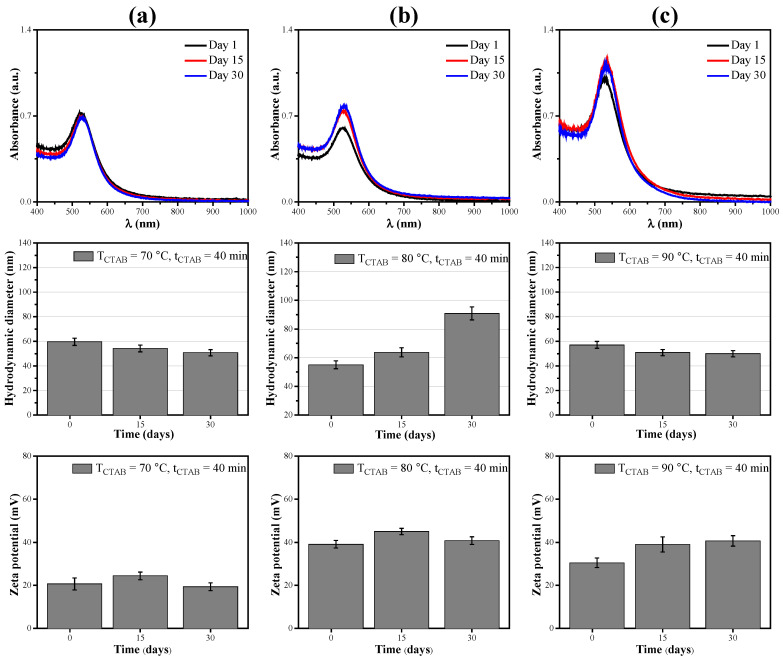
Temporal monitoring of UV-Vis absorbance spectra, hydrodynamic diameters, and zeta potential values for samples (**a**) H13b, (**b**) H14b and (**c**) H15b.

For samples H13b, H14b, and H15b (Figure 6), the UV–Vis spectra remain essentially unchanged over 30 days, with no pronounced peak broadening or loss of the LSPR band, which suggests preservation of the plasmonic signature and absence of major aggregation under the tested conditions. DLS measurements show a modest decrease in hydrodynamic diameter from ~60 nm to ~50 nm over time, which may reflect progressive equilibration/reorganization of the CTAB interfacial layer and the associated electrical double layer (or the redistribution of weak, soft aggregates) rather than a change in the metallic core size. The measured zeta potentials remain positive and vary within a relatively narrow range of approximately +20 to +25 mV, indicating sustained electrostatic stabilization of the colloid [21]. Overall, these trends are consistent with a stable CTAB-stabilized dispersion, with any slow evolution plausibly associated with interfacial restructuring and/or ripening-type processes [19].

### 3.5. TEM Analysis of CTAB Modified AuNPs

**Figure 7:**
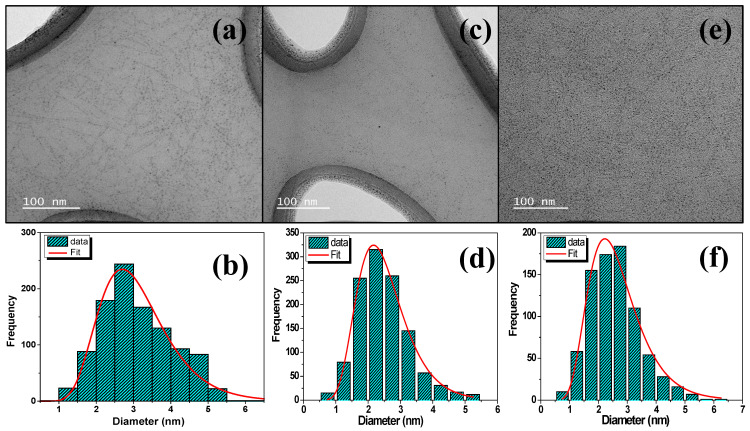
(**a**,**c**,**e**) Representative TEM images and (**b**,**d**,**f**) PSD histogram for H13, H14, and H15 samples. PSD was obtained from 20 to 25 micrographs per sample (scale bars 50–200 nm), totaling ~800–1200 particles measured using ImageJ.

In Figure 7, the most representative images for these three samples are shown. The analysis of H13, H14, and H15 samples shows that the AuNPs have a well-defined size distribution centered around 2 to 3 nm. The PSD histogram follows a log-normal shape, as observed in the red fit. In the analysis conducted, it was observed that samples H13–H15 exhibited a polydispersity index (PDI) of less than 0.2, indicating that the samples have a narrow metallic core-size distribution by TEM [22]. It is worth mentioning that it is very important to have a good statistical count to model the distribution trend and thus obtain the corresponding statistical data. For all the samples, the PDI of 6–7% indicates good uniformity in the AuNPs size distribution. In Table 3, the morphological parameters obtained from the fitting of the PSD histogram are observed.

It is important to clarify the apparent discrepancy between the ultrasmall metallic core diameters determined by TEM (2–3 nm) and the significantly larger hydrodynamic diameters obtained by DLS (≈97–110 nm). While TEM provides direct visualization of the metallic Au core in the dried state, DLS measures the hydrodynamic diameter of the particles in suspension, which includes not only the metallic core but also the surrounding CTAB surfactant layer, the associated electrical double layer, and any dynamic micellar assemblies present in solution. CTAB is known to adsorb onto Au surfaces, forming organized monolayer or bilayer structures and, depending on concentration and temperature, may also generate micelle-like aggregates around AuNPs [9]. These soft organic shells and hydration layers significantly increase the apparent size measured by DLS. Therefore, the larger hydrodynamic diameters do not contradict the TEM observations but rather reflect the effective colloidal entity in solution.

In terms of the aggregation state in suspension, it is important to note that a hydrodynamic diameter of ~97–110 nm does not necessarily imply that the metallic Au cores are aggregated into compact ~100 nm solid clusters. DLS is intensity-weighted and highly sensitive to the effective colloidal entity in liquid, such that soft interfacial layers and CTAB-associated micellar structures can dominate the apparent size. The consistently high positive zeta potentials measured for the CTAB-stabilized samples (typically >+35 mV and reaching >+40 mV), together with the temporal stability of the LSPR band and the absence of strong time-dependent increases in DLS diameter, support strong electrostatic stabilization and do not indicate extensive aggregation under the tested conditions.

A clear distinction must be made between the PDI values obtained from DLS measurements and those derived from TEM-based size analysis. The PDI determined by DLS reflects the variability of hydrodynamic diameters in colloidal suspension, capturing the effective particle entity in liquid (metallic core plus surfactant layer, hydration shell, and electrical double layer). Conversely, the PDI calculated from TEM images corresponds solely to the distribution of metallic core sizes measured under dried conditions. Consequently, these indices quantify different physical aspects of the system and should not be directly compared; instead, they provide complementary information on colloidal dispersion versus intrinsic core-size uniformity. Moreover, the consistently high positive zeta potentials measured for the CTAB-stabilized samples (typically > +35 mV and reaching > +40 mV) support strong electrostatic stabilization, indicating that the larger DLS diameters primarily reflect the CTAB corona/micellar contribution rather than extensive aggregation.

**Figure 8:**
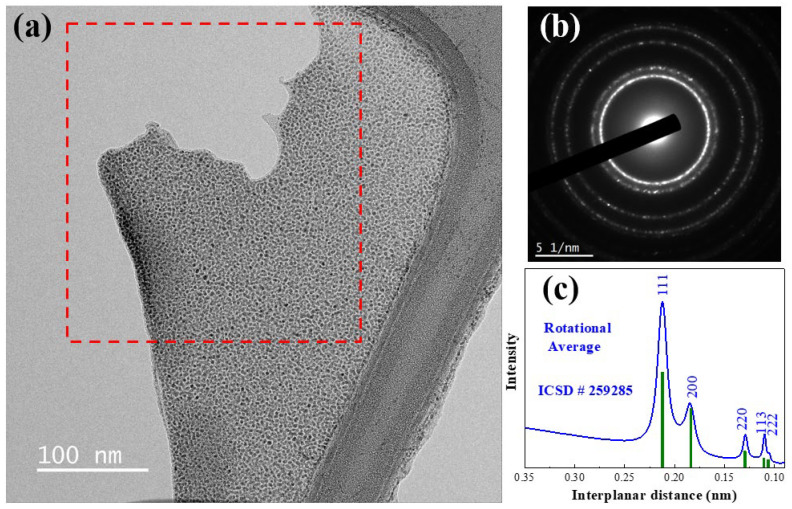
(**a**) Representative TEM image for H13 sample, the red inset indicates the region where the SAED pattern (**b**) was taken, (**c**) is the rotational average pattern generated for 3 nm Au phase found in the H13 sample. The vertical green lines indicate the reference Bragg peak positions of the Au phase (ICSD #259285).

As shown by TEM images, they all have the same narrow and ultrasmall core size. In that sense, to determine the Au phase presented in the NPs, the SAED pattern was taken to a representative image of H13 sample, see Figure 8a,b. The rotational average pattern, in Figure 8c, was indexed using the ICSD #259285 assigned to Au crystal structure (Fm-3m space group). The interplanar distance of the first ring (111) corresponded to the Au phase and was found to be 0.22 nm.

The results discussed in Section 3.1, Section 3.2, Section 3.3, Section 3.4 and Section 3.5 establish a clear structure–stability relationship for chemically synthesized AuNPs stabilized by CTAB, where controlled surfactant dissolution governs nanoparticle size, monodispersity, and electrostatic stabilization. In this inorganic route, colloidal stability is primarily dictated by the presence of a well-defined cationic surfactant layer, leading to ultrasmall and narrow core size distributions with reproducible physicochemical properties.

In contrast, biosynthesis routes introduce a fundamentally different stabilization and growth mechanism, driven by a complex mixture of organic molecules with diverse functional groups and reduction kinetics. Unlike CTAB, plant-derived metabolites provide heterogeneous and dynamic capping environments that may promote broader size distributions, multiple growth regimes, and distinct surface chemistries. To elucidate these differences and assess their impact on nanoparticle formation and stability, the following sections focus on the optical, hydrodynamic, electrostatic, and morphological characterization of AuNPs biosynthesized using *Erythroxylum coca* leaf extracts. This comparison enables a direct evaluation of how inorganic surfactant-mediated synthesis and organic extract-mediated biosynthesis differentially influence nanoparticle properties.

Importantly, the CTAB-mediated and extract-mediated syntheses differ not only in the nature of the stabilizing/capping environment but also in the kinetic and thermodynamic conditions that govern nucleation and growth. In the inorganic route, NaBH_4_ is a strong reducing agent, and the reaction is conducted at elevated temperature, conditions that favor rapid Au^3+^ reduction, burst nucleation, and early growth quenching, which are consistent with ultrasmall metallic cores observed by TEM. In contrast, the biosynthetic route proceeds at room temperature over extended times using a chemically heterogeneous mixture of mild reductants, which can yield slower reduction kinetics, lower instantaneous nucleation density, and prolonged growth/aggregation and secondary anisotropic processes. Consequently, the size and stability differences reported here should be interpreted as arising from the interplay between (i) reduction kinetics and reaction temperature/time and (ii) the distinct interfacial stabilization mechanisms (organized cationic CTAB layer vs. heterogeneous organic corona), rather than being attributed solely to “surfactant vs. organic coating”.

### 3.6. UV-Vis Analysis of Biosynthesized Samples

**Figure 9:**
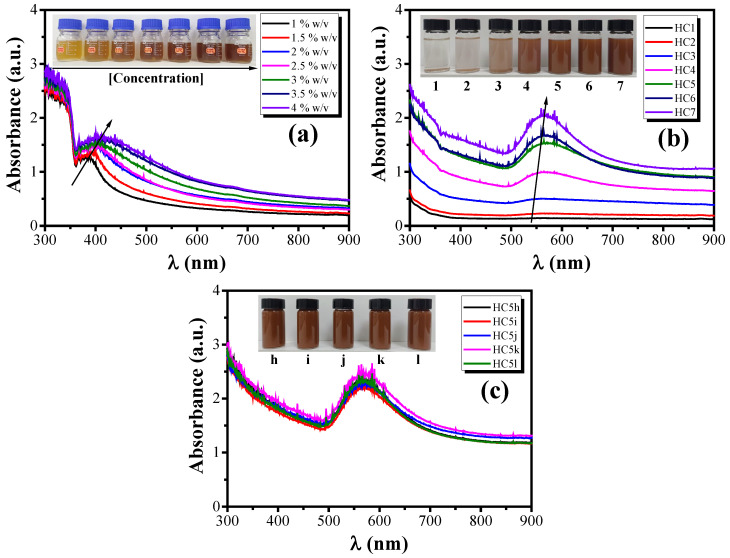
(**a**) UV–Vis spectra of coca leaf extracts prepared at different concentrations (% *w*/*v*), showing an increase in absorbance intensity and a progressive red shift with increasing extract concentration. (**b**) UV–Vis spectra of AuNPs synthesized using extracts at different concentrations (HC1–HC7), where the appearance of the LSPR band in the range of ~550–580 nm confirms AuNP formation and reveals concentration-dependent optical behavior. (**c**) UV–Vis spectra of five independent replicates of the optimal sample (HC5), demonstrating a consistent LSPR band centered around ~560 nm, indicative of high reproducibility and uniform optical properties of the biosynthesized AuNPs. In panels (**a**,**b**), the horizontal arrow labeled [Concentration] indicates increasing extract concentration from left to right, while the arrows on the spectra indicate the direction of the concentration-dependent spectral changes, including increased absorbance intensity and band red shift. In panel (**b**), the numbers 1–7 shown on the vial photographs correspond to samples HC1–HC7, respectively. In panel (**c**), the letters h–l identify the five independent replicates of sample HC5, namely HC5h, HC5i, HC5j, HC5k, and HC5l, respectively.

The UV–Vis spectra of the coca leaf extracts exhibit a set of absorption bands whose maxima progressively shift from ~380 nm to ~420 nm as the extract concentration increases. This shift toward longer wavelengths (red shift) suggests an increase in optical density associated with a higher abundance of phenolic metabolites, flavonoids, and other aromatic compounds that absorb in this region. The concentration-dependent increase in absorbance is consistent with a larger content of chromophoric molecules, indicating that the more concentrated extract not only displays stronger optical intensity but also a chemically richer composition in functional groups capable of acting as reducing and stabilizing agents. These chemical features are expected to directly impact the subsequent biosynthesis of AuNPs (Figure 9a).

Although *Erythroxylum coca* is known to contain tropane alkaloids (e.g., cocaine-type derivatives), it is important to clarify that these molecules are not necessarily the primary electron donors responsible for Au^3+^ → Au^0^ reduction under the present aqueous biosynthesis conditions. Tropane alkaloids are tertiary amines with ester functionalities and aromatic groups, which are more plausibly involved in coordination/ion-pair interactions with AuCl_4_^−^, adsorption at the metal–solution interface, and surface passivation/facet-selective binding that can influence growth pathways and colloidal stability, rather than acting as strong reductants. In contrast, polyphenolic and flavonoid constituents, which are commonly present in plant extracts and exhibit higher reducing capacity, are more likely to dominate the reduction step, while alkaloids and other metabolites contribute to the formation of a heterogeneous organic corona that modulates nucleation, growth, and (in a minor fraction) anisotropic evolution. Because no FTIR/HPLC profiling was performed on the extract in this study, these assignments are presented as physicochemically plausible interpretations rather than compound-specific proof; future work will couple extract fingerprinting (FTIR/HPLC/LC–MS) with model-compound controls to identify the dominant reducing/capping species.

Figure 9b shows the UV–Vis spectra of AuNPs synthesized using each extract concentration. From sample HC4 onward, the characteristic LSPR band typical of AuNPs becomes clearly evident, with maxima in the ~550–580 nm range. The slight red shift in the LSPR maximum as the extract concentration increases indicates changes in nanoparticle size and/or size distribution, since red shifts are commonly associated with larger particles and/or an increase in the local dielectric environment. In addition, the variations in absorbance intensity are consistent with enhanced reducing efficiency at higher extract concentrations, which promotes the formation of a larger number of AuNPs. Overall, these results confirm that the plant extract concentration directly influences both the formation and the optical properties of the biosynthesized AuNPs.

Figure 9c presents five independent replicates of the optimal sample (HC5), each displaying a consistent LSPR band centered at approximately 560 nm. The nearly identical overlap in both peak position and absorbance intensity demonstrates high reproducibility of the biosynthesis process. Quantitative analysis of these replicates is summarized in Table 4, which compiles the statistical parameters obtained from five independent preparations (n = 5), including plasmonic characteristics, *D*_H_, and |ζ|. The mean maximum absorbance reached 2.34 ± 0.11 a.u., while the average LSPR peak position was 569.6 ± 1.7 nm, indicating minimal spectral variation between batches. Additionally, the full width at half maximum (FWHM) remained highly consistent (118 ± 1.2 nm), reflecting negligible changes in peak broadening and suggesting a stable nanoparticle size distribution. This statistical consistency supports the high reproducibility of the biosynthesis protocol and indicates that the optimized conditions produce AuNPs with uniform optical characteristics and stable colloidal behavior.

### 3.7. Dynamic Light Scattering and Zeta Potential

**Figure 10:**
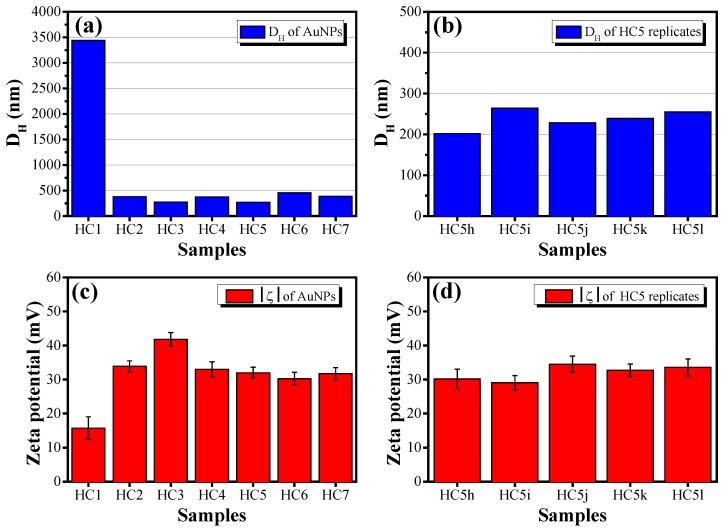
DLS and zeta potential analysis of biosynthesized AuNPs. (**a**) Hydrodynamic diameter of AuNPs synthesized using different extract concentrations (HC1–HC7). (**b**) Hydrodynamic diameter of five independent replicates of the optimal sample (HC5). (**c**) Zeta potential of AuNPs synthesized with different extract concentrations. (**d**) Zeta potential of the HC5 replicates, demonstrating the reproducibility and colloidal stability of the biosynthesized AuNPs.

Figure 10a shows the hydrodynamic diameter of the AuNPs, which remains within a general range of approximately 270–390 nm, indicating a relatively stable size distribution. However, sample HC1 exhibits an anomalously high hydrodynamic diameter of 3341 nm, clearly evidencing severe aggregation. This behavior is attributed to the insufficient amount of coca leaf extract under this condition, which was unable to adequately stabilize the AuNPs, thereby allowing particle agglomeration and leading to a drastic increase in the measured hydrodynamic size.

Figure 10b presents the hydrodynamic diameter distribution obtained for the HC5 sample replicates. The measured values range between 202 and 264 nm, demonstrating good agreement among independent biosynthesis experiments. Quantitative analysis (Table 4) yielded an average hydrodynamic diameter of 237.6 ± 24.3 nm, indicating moderate dispersion within the colloidal system while maintaining a consistent particle population across replicates. This relatively narrow size variation confirms that the optimized synthesis conditions promote reproducible nanoparticle formation and stable colloidal behavior.

Figure 10c shows the zeta potential values measured for the biosynthesized AuNPs under different extract concentrations. Most samples exhibit zeta potentials within the 30–40 mV range, which is generally associated with strong electrostatic stabilization of colloidal AuNPs. The only exception corresponds to sample HC1, which displays a significantly lower zeta potential value of 16 mV, indicating reduced surface charge and decreased colloidal stability. This behavior can be attributed to the low concentration of coca leaf extract, which likely provided an insufficient amount of stabilizing phytochemicals to effectively passivate the nanoparticle surface, thereby promoting aggregation.

The reproducibility of the optimized synthesis conditions is further confirmed in Figure 10d, where the HC5 replicates exhibit zeta potential values consistently close to 32 mV. Statistical analysis summarized in Table 4 yields a mean zeta potential of 32.2 ± 2.6 mV, indicating a highly stable colloidal system with minimal variation in surface charge between independent preparations. Together with the optical reproducibility observed in the UV–Vis spectra, these results confirm that the optimized HC5 biosynthesis protocol provides reliable control over nanoparticle formation, producing AuNPs with uniform physicochemical properties and good colloidal stability.

### 3.8. TEM Analysis of Biosynthesized Samples

**Figure 11:**
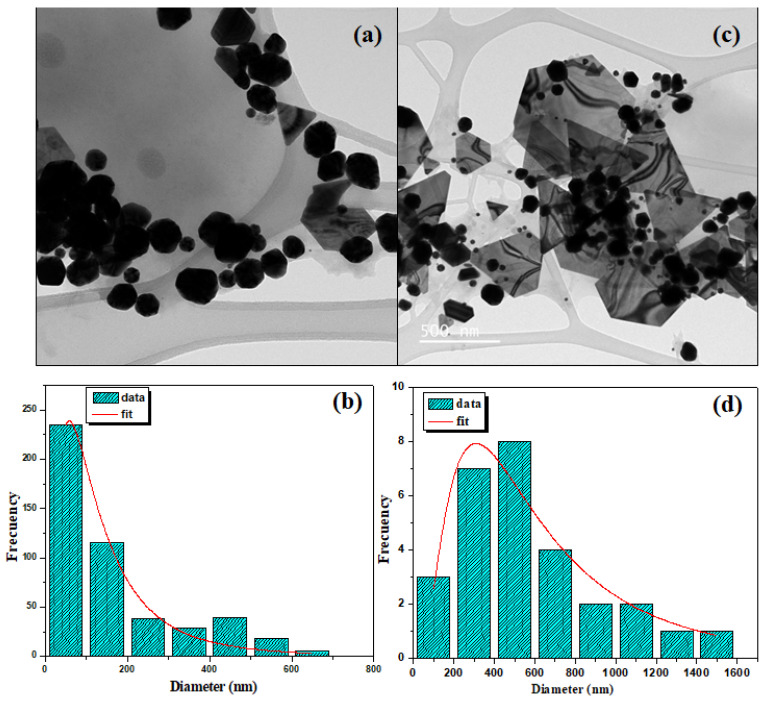
(**a**,**c**) Representative TEM images of HC5 sample and (**b**,**d**) PSD histogram. Bar scale of 200 nm (**a**).

The size distribution of the AuNPs (representative HC5 sample) synthesized using coca leaf extract exhibits a lognormal behavior [5], which is characteristic of biosynthesis processes in which nucleation and growth occur in a non-uniform manner [23]. This type of distribution has been widely reported in green synthesis approaches due to the complex nature of plant extracts, which contain multiple biomolecules (biological substrates) with different reducing and stabilizing capacities. However, the asymmetry of the distribution toward larger diameters reveals that a minor fraction of the system continued to grow after the main nucleation event or underwent partial aggregation processes, resulting in larger circular particles. This behavior suggests the coexistence of a dominant rapid nucleation regime with subsequent differential growth events under kinetic control. The heterogeneous stabilization of the AuNPs indicates that the kinetic growth process is influenced by the presence of organic components in the extract [24,25]. The observed polydispersity is consistent with the use of plant extracts, in which alkaloids, polyphenols, and other secondary metabolites coexist and interact differently with metallic nuclei. In particular, coca leaf extract may generate regions with a high density of stabilizing agents, favoring the formation of small AuNPs, while in other regions the organic capping is insufficient, allowing continued growth or aggregation of previously formed particles (Figure 11).

The size distribution obtained from the analysis of 491 AuNPs was adequately fitted to a lognormal model (R^2^ = 0.9699), confirming a behavior typical of biosynthesis processes mediated by plant extracts. Using the log-normal fit ($x_{c}=113.99$ nm, median; w=0.82, log-space standard deviation), the mean diameter was estimated to be ~160 nm. The calculated PDI ≈ 0.96 indicates a broad size distribution, suggesting that rapid nucleation processes occurred simultaneously with subsequent secondary growth events or partial aggregation during synthesis. The TEM morphological analysis revealed the coexistence of two distinct populations of AuNPs synthesized using coca leaf extract: a predominant population of circular particles (n = 491) and a minor fraction of triangular microcrystal type particles (n = 28). In both cases, the size distribution was adequately fitted to a lognormal model.

In contrast, the population of triangular particles exhibited a considerably broader dimensional range, approximately between 100 and 1500 nm, with a higher frequency within the 300–600 nm interval. The presence of a pronounced tail toward sizes above 1000 nm suggests the occurrence of prolonged growth processes or oriented aggregation in a limited fraction of the system. The low relative frequency of this morphology (n = 28) indicates that it does not correspond to the dominant primary nucleation mechanism, but rather to secondary anisotropic growth events. The formation of triangular AuNPs is typically associated with preferential growth along specific crystallographic planes, promoted by the selective adsorption of organic molecules onto particular facets [26].

The marked dimensional difference between both populations supports the hypothesis that the biosynthesis process involves at least two growth regimes: (i) a rapid and dominant nucleation stage that gives rise to nanometric spherical particles, and (ii) a secondary anisotropic growth process responsible for the formation of larger triangular particles. This behavior has been reported in multiple green synthesis approaches, where the chemical complexity of the plant extract introduces variability in reduction kinetics and in the efficiency of colloidal stabilization. From a functional perspective, the predominance of small AuNPs is advantageous for applications requiring high specific surface area, such as catalysis and sensing. On the other hand, the presence of larger triangular particles may impart distinct plasmonic properties, particularly red-shifts toward longer wavelength spectral regions associated with anisotropic resonance modes [27,28]. Overall, the results demonstrate that biosynthesis mediated by coca leaf extract produces a polydisperse system predominantly composed of nanometric spherical morphologies, along with a secondary fraction of larger anisotropic structures.

**Scheme 3:**
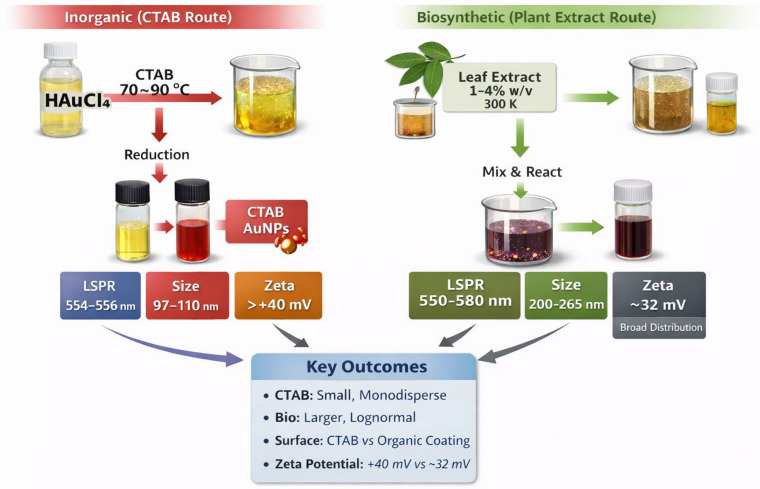
Comparative AuNP synthesis routes via (**left**) CTAB-mediated inorganic synthesis and (**right**) *Erythroxylum coca* extract-mediated biosynthesis, highlighting key process variables including reducing environment (NaBH_4_ vs. plant metabolites), reaction temperature and duration (short high-temperature reduction vs. 24 h at room temperature), and interfacial stabilization mechanism (organized cationic CTAB layer vs. heterogeneous organic corona), together with the resulting physicochemical signatures (LSPR, hydrodynamic size, and zeta potential).

To consolidate the comparative framework developed throughout this work, Scheme 3 provides a final side-by-side schematic of the inorganic (CTAB-mediated) and biosynthetic (leaf-extract-mediated) AuNP synthesis routes, highlighting the key processing variables and the resulting quantitative physicochemical signatures (LSPR position, hydrodynamic size, and zeta potential) that distinguish both pathways.

## 4. Conclusions

This work provides a quantitative comparison between CTAB-mediated inorganic synthesis and *Erythroxylum coca* extract-mediated biosynthesis of AuNPs, showing how synthesis pathways influence optical response, effective colloidal size, surface charge, and stability. In the CTAB route, AuNPs exhibited LSPR bands at ~554–556 nm and high positive zeta potentials (typically >+35 mV and reaching >+40 mV), indicating strong electrostatic stabilization and stable dispersions over the monitoring period. TEM revealed ultrasmall metallic cores (≈2–3 nm), whereas DLS yielded hydrodynamic diameters of ~97–110 nm. This discrepancy is expected because DLS measures the effective entity in suspension (metal core plus CTAB corona, hydration/electrical double layer, and possible micellar contributions), and the consistently high zeta potentials suggest that the larger DLS sizes primarily reflect interfacial corona effects rather than extensive aggregation. In contrast, extract-mediated biosynthesis produced AuNPs with LSPR bands in the ~550–580 nm range and larger hydrodynamic diameters, consistent with heterogeneous organic capping by plant-derived metabolites and broader growth regimes. Low extract concentration led to reduced surface charge and pronounced aggregation, whereas optimized conditions produced reproducible colloids with stable |ζ| values and consistent optical signatures. Notably, the observed differences in effective size and growth behavior also reflect substantial differences in reduction kinetics and reaction temperature/time between the two routes, which act together with the interfacial capping environment to determine nucleation density and growth regimes. Overall, these results indicate a mechanistic distinction between (i) electrostatic stabilization governed by an organized cationic CTAB interfacial layer in the inorganic route and (ii) heterogeneous organic corona capping in the biosynthetic route, where mixed functional groups may provide combined electrostatic/steric stabilization and can promote secondary anisotropic growth in a minority population. While the biosynthetic route reduces reliance on synthetic surfactants such as CTAB, the present study was focused exclusively on physicochemical characterization; therefore, no direct biological assays were performed to quantitatively assess cytocompatibility. Dedicated in vitro studies (e.g., MTT or LDH assays) will be necessary in future work to rigorously evaluate and compare the biological responses associated with each synthesis pathway. These findings emphasize the critical role of synthesis conditions in tailoring AuNP physicochemical properties and provide a quantitative framework for selecting synthetic strategies according to targeted applications.

## Figures and Tables

**Table 1:** CTAB dissolution time and temperature for each synthesized sample.

Sample	Dissolution Time of CTAB (min)	Dissolution Temperature of CTAB (°C)
H13	20	70
H14	20	
H15	20	80
H13b	40	
H14b	40	90
H15b	40	

**Table 2:** Parameters used for the biosynthesis of AuNPs.

Sample	Precursor Volume (mL)	Extract Concentration (% *w*/*v*)	Extract Volume (mL)	Reaction Time (h)
HC1	20	1.0	4	24
HC2	20	1.5	4	24
HC3	20	2.0	4	24
HC4	20	2.5	4	24
HC5	20	3.0	4	24
HC6	20	3.5	4	24
HC7	20	4.0	4	24

**Table 3:** Morphological parameters obtained from the fitting of the particle size distribution histogram. *D* is the particle diameter (nm), σ is the standard deviation, and n is the number of particles.

Sample	*D*	σ	PDI	n
**H13**	3.05(8)	0.17	0.06	1032
**H14**	2.39(2)	0.16	0.07	1186
**H15**	2.51(5)	0.18	0.07	798

**Table 4:** Statistical parameters obtained from five independent HC5 biosynthesis replicates (n = 5), including λ_max_, FWHM, *D*_H_, and |ζ|. The results demonstrate the reproducibility of the optimized biosynthesis conditions. pH of the suspensions = 6.5.

HC5 Replicates	Replica Number	Absorbance (a.u)	λ_max_ (nm)	FWHM (nm)	*D*_H_ (nm)	|ζ| (mV)
HC5h	1	2.4	569	118	202	30
HC5i	2	2.2	570	118	264	29
HC5j	3	2.3	571	119	228	35
HC5k	4	2.5	571	119	239	33
HC5l	5	2.3	567	116	255	34
	**Mean**	2.34	569.6	118	237.6	32.2
	**Standard deviation**	0.11	1.7	1.2	24.3	2.6

## Data Availability

The original contributions presented in this study are included in the article. Further inquiries can be directed to the corresponding author.
